# ROS-mediated NLRP3 inflammasome activation participates in the response against *Neospora caninum* infection

**DOI:** 10.1186/s13071-020-04331-8

**Published:** 2020-09-05

**Authors:** Lu Li, Xiao-Cen Wang, Peng-Tao Gong, Nan Zhang, Xu Zhang, Shan Li, Xin Li, Shao-Xiong Liu, Xiao-Xu Zhang, Wei Li, Jian-Hua Li, Xi-Chen Zhang

**Affiliations:** 1 Key Laboratory of Zoonosis Research, Ministry of Education, College of Veterinary Medicine, Jilin University, Changchun, 130062 PR China; 2 Heilongjiang Key Laboratory for Zoonosis, College of Veterinary Medicine, Northeast Agricultural University, Harbin, 150030 PR China

**Keywords:** *Neospora caninum*, NLRP3 inflammasome, Reactive oxygen species, Pyrogallol, Host defense

## Abstract

**Background:**

*Neospora caninum* is an obligate intracellular protozoan that causes neosporosis, *N. caninum* infection is a major cause of abortion in cattle worldwide. Currently, specific treatment for neosporosis is not available. The NOD-like receptor family pyrin domain containing 3 (NLRP3) inflammasome is a cytoplasmic protein complex that plays an important role in host defense against *N. caninum* infection, but the underlying mechanisms are poorly understood.

**Methods:**

The reactive oxygen species (ROS) inhibitor and the ROS inducer, wild-type (WT) and NLRP3-deficient peritoneal macrophages or mice were used to investigate the role of ROS in NLRP3 inflammasome activation and controlling parasite burdens. ROS production, cell death and cell viability, production of inflammasome-mediated IL-1β or IL-18, cleavage of caspase-1 and NLRP3 expression, as well as parasite burdens were detected.

**Results:**

*In vitro*, *N. caninum* induced ROS generation in a dose-dependent manner in peritoneal macrophages. The pretreatment of ROS inhibitor *N*-acetyl-l-cysteine (NAC) significantly attenuated *N. caninum*-induced ROS production, LDH release, IL-1β secretion and NLRP3 expression, whereas *N. caninum* proliferation was notably increased. In contrary, the ROS inducer pyrogallol (PG) significantly enhanced ROS production and NLRP3 inflammasome activity and decreased the parasite burden in *N. caninum*-infected peritoneal macrophages. NADPH-dependent ROS-mediated NLRP3 inflammasome activation induced by *N. caninum* can also be confirmed by using the NADPH oxidase inhibitor diphenyleneiodonium chloride (DPI). However, the NAC or DPI pre-treatment or PG treatment did not significantly alter *N. caninum*-induced inflammasome activities and parasite proliferation in *Nlrp3*^*−/−*^ peritoneal macrophages. *In vivo*, IL-18 releases in serum and parasite burdens in peritoneal exudate cells were significantly increased in PG-treated WT mice after infection with *N. caninum*; however, IL-18 productions and parasite burdens were not changed in PG-treated *Nlrp3*^*−/−*^ mice. Furthermore, PG treatment in WT mice infected with *N. caninum* significantly decreased the mortality, weight loss and parasite burdens in tissues and histopathological lesions.

**Conclusions:**

*Neospora caninum-*induced NADPH-dependent ROS generation plays an important role in NLRP3 inflammasome activation and controlling parasites. The ROS inducer PG can control *N. caninum* infection mainly by promoting NLRP3 inflammasome activation. ROS-mediated NLRP3 inflammasome axis can be a potential therapeutic target for neosporosis.
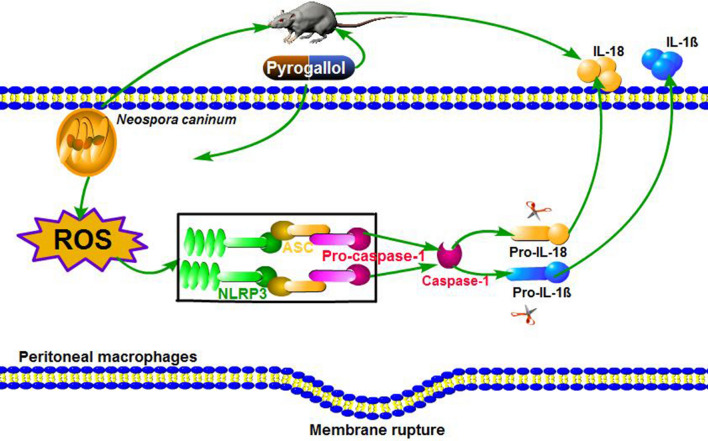

## Background

*Neospora caninum* is an intracellular protozoan parasite [1–3], mainly causes neuromuscular disorders in canines, and abortion and stillbirth in ruminants. Neosporosis is considered as one of the main infectious causes of abortion in cattle, as well as stillbirth and the birth of weak calves, leading to a significant economic burden on the beef and dairy industries worldwide [4]. Currently, neither effective vaccines nor drugs have been developed for the prevention and treatment of neosporosis due to the elusive anti-*N. caninum* immune mechanisms in the hosts [5, 6]. *Neospora caninum* has three infectious stages (tachyzoites, bradyzoites and sporulated oocysts). During acute *N. caninum* infection, tachyzoites disseminate *via* the blood stream and lymphatic system, and trigger the innate and the adaptive immune response [7]. It is reported that the pathogenesis of acute neosporosis is closely related to the rapid proliferation of *N. caninum* tachyzoites in cells [8]. The host innate immune response plays a critical role in recognizing pathogen and inhibiting initial parasite replication, subsequently mediating appropriate adaptive immune response to promote the host resistance to this infection [9, 10]. The pattern-recognition receptors (PRRs) of innate immune cells are activated in *N. caninum* infection, such as TLR2 [11] and TLR3 [12] from the Toll-like receptors family (TLRs), NOD2 [9] and NLRP3 [8] from the NOD-like receptors family (NLRs). These PRRs can promote host defense to *N. caninum* infection or contribute to the pathogenesis. We have previously found that the sensor NLRP3 was upregulated and NLRP3 inflammasome was activated in *N. caninum*-infected peritoneal macrophages (PMs), and this pathway functioned in the elimination of *N. caninum* [8]. However, its molecular activation mechanisms need to be further explored.

The NLRP3 inflammasome is a multimeric protein complex found in the cytosol. The sensor NLRP3 interacts with the adaptor molecule ASC (apoptosis-associated speck-like protein), and the caspase recruitment domain (CARD) of ASC binds to the CARD domain of caspase-1 *via* protein-protein domain interactions to form the NLRP3 inflammasome [13, 14]. Inflammasome activation leads to the activation of caspase-1, cleavage of active interleukin (IL)-1β and IL-18, and caspase-1-dependent cell death (known as pyroptosis) [15]. The NLRP3 inflammasome is activated by various pathogen-associated molecular patterns (PAMPs) including microbial nucleic acids or proteins, and host-derived danger-associated molecular patterns (DAMPs) such as uric acid crystals [16–18]. Multiple cell-signaling mechanisms can participate in NLRP3 activation, including the release of reactive oxygen species (ROS), potassium efflux, lysosomal damage, and mitochondrial destabilization or damage [19]. Additionally, NLRP3 could be directly regulated by ROS [20].

ROS generation has been identified as a key factor in NLRP3 inflammasome activation in parasitic infections by those such as *Plasmodium falciparum* [21], *Trypanosoma cruzi* [22], and *Toxoplasma gondii* [13, 23]. ROS has been implicated in the regulation of many important cellular events, including proliferation, differentiation, immune response, cell growth, and cell survival [24, 25]. Our previous study showed that *N. caninum* tachyzoites can induce NADPH oxidase-dependent ROS generation in canine polymorphonuclear neutrophils [26]. As the NADPH oxidase-dependent ROS generation contributes to Nalp3 inflammasome activation [27], it is therefore conceivable that *N. caninum*-infected PMs undergo NLRP3 inflammasome activities due to ROS production. Nevertheless, whether ROS is involved in NLRP3 inflammasome activation during *N. caninum* infection and how this axis controls the parasite burden, remain to be clarified. To the best of our knowledge, this is the first study that investigates ROS-mediated NLRP3 inflammasome activation and its role in controlling *N. caninum* replication in *N. caninum*-infected PMs, and assesses the potential of ROS inducer pyrogallol (PG) in activating ROS-dependent NLRP3 inflammasome and eliminating parasites *in vitro* and *in vivo*.

## Methods

### Animals

Wild-type (WT) female C57BL/6 mice (6–8 weeks-old) were purchased from the Changsheng Experimental Animal Center (Benxi, China), and NLRP3-deficient female mice backcrossed onto the C57BL/6 genetic background were obtained from the Jackson Laboratory (Bar Harbor, ME, USA). The mice were maintained in specific pathogen-free conditions and housed in isolator cages in the animal house of the Laboratory Animal Center of Jilin University. Drinking water and food were supplied *ad libitum*.

### Parasites and cell culture

*Neospora caninum* (Nc-1 isolate) tachyzoites were maintained by serial passages in Vero cells, which were cultured in Roswell Park Memorial Institute (RPMI)-1640 medium (Life Technologies, Grand Island, NY, USA) supplemented with 2% heat-inactivated fetal bovine serum (FBS; Biological Industries, Ltd., Beit HaEmek, Israel), 2 mM l-glutamine, 100 U/ml penicillin, and 100 μg/ml streptomycin (all from Life Technologies), and they were incubated at 37 °C under 5% CO_2_. *Neospora caninum* tachyzoites were harvested when 80% of Vero cells were lysed, parasites and Vero cell debris were then treated with a 27 gauge needle, and Vero cell debris were removed by gradient density centrifugation with a 40% Percoll (GE Healthcare, Uppsala, Sweden) solution (v/v), centrifuged at 1500× *g* for 30 min, then the pellet of *N. caninum* tachyzoites was harvested and washed twice with RPMI-1640 (centrifuged at 900× *g* for 10 min) [8], and the *N. caninum* concentration was measured by using a hemocytometer. Female WT and *Nlrp3*^*−/−*^ mice were inoculated intraperitoneally with 3 ml of 5% thioglycolate medium (BD Biosciences, New Zealand, USA) for 4 days, and the mice were humanely euthanized [28] and soaked with 75% alcohol for 10 min, PMs were obtained by flushing the peritoneal cavity twice with cold phosphate-buffered saline (PBS), then the cell suspension was centrifuged at 1000× *g* for 10 min, and washed twice with PBS [29]. The PMs were cultured in R-10% medium (comprising RPMI supplemented with 10% heat-inactivated FBS, 2 mM l-glutamine, 100 U/ml penicillin, and 100 μg/ml streptomycin) for at least 12 h.

### Experimental infection and stimulation

PMs were isolated from WT or *Nlrp3*^*−/−*^ mice and infected with *N. caninum* at a multiplicity of infection (MOI) of 3:1, 2:1 or 1:1 (parasite: cell) in R-1% medium for 2, 4 or 8 h, then PMs were washed twice with PBS to remove non-adhered parasites. In the experimental group, cells were pre-treated with the ROS inhibitor NAC for 1 h (5 mM; Selleck, Shanghai, China), or the NADPH oxidase inhibitor diphenyleneiodonium chloride (DPI, 10 μM; Selleck) for 2 h [30]. Additionally, at 2, 4 or 8 h post-infection (pi), the medium was changed to fresh medium with or without the ROS inducer pyrogallol (PG; Selleck). PMs cultured with the equivalent volume of R-1% medium was used as a negative control. Adenosine triphosphate (ATP) is a NLRP3 inflammasome inducer [31], so PMs in positive control group were pre-treated with *N. caninum* and then stimulated with 5 mM ATP (Sigma-Aldrich, Shanghai, China) for 30 min. At 5, 24 or 36 h post-infection, cells and supernatants were collected for subsequent experiments, as described below.

### Anti-*N. caninum* activity of PG *in vivo*

WT and *Nlrp3*^*−/−*^ female mice were used to establish an animal model of acute *N. caninum* infection. The two kinds of mice were each randomly divided into three groups: PBS-treated mice; *N. caninum*-infected mice; and *N. caninum*-infected with PG-treated mice (Sigma-Aldrich). Each group consisted of 5 mice. PG was dissolved in PBS. The mice were intraperitoneally injected with 2.5 × 10^7^ *N. caninum* tachyzoites or the same volume of PBS. From day 2 pi, mice in the infection groups were intraperitoneally injected daily with 0.5 ml of 75 mg/kg PG [32, 33] for 5 days, and the remaining mice were administered the same volume of PBS. On day 8, peritoneal exudate cells were harvested as previously described [34], and serum, heart, liver, spleen, lung, kidney, and brain samples were collected. Fragments of these tissues were immediately fixed in 10% formalin buffer solution and routinely processed for paraffin embedding, and histopathological changes were examined with H&E staining under light microscopy.

### Intracellular ROS detection

Intracellular ROS generation in PMs (MOI of 3:1 or 1:1) was measured using the 2,7 dichlorofluorescein diacetate (DCFH-DA) (Sigma-Aldrich). PMs were plated in triplicate in 96-well plates at a density of 5 × 10^4^ cells/well and 6-well plates at a density of 3 × 10^6^ cells/well. PMs were pre-treated with NAC (for 1 h), or DPI (for 2 h), then incubated with *N. caninum* for 2 h, washed twice with RPMI 1640 to remove non-adhered parasites, then cultured in fresh R-1% medium for 3 h. In the PG treatment group, fresh R-1% medium was added with PG (the dilutions included concentrations from 15, 30, 60 or 80 μM). PMs were stimulated with zymosan (1 mg/ml; Sigma-Aldrich) as a positive control. After incubation, the supernatant was removed, and the PMs were incubated with the DCFH-DA for 20–30 min at 37 °C, followed by 3 washes with FBS-free RPMI in the dark. PMs in 96-well plates were assessed with a multifunctional fluorescence microplate reader (M2e, Molecular Devices, Sunnyvale, CA, USA), using 488 nm emission and 525 nm excitation. And PMs collected from 6-well plates were analyzed using an FACSAria flow cytometer (BD Biosciences).

### Lactate dehydrogenase (LDH) release assay

Pyroptosis was analyzed by detecting the activity of LDH released into the cell supernatants (collected from 3 × 10^6^ cells) at different treatment for 24 or 36 h using an LDH assay (Roche Diagnostics, Mannheim, Germany) according to the manufacturer’s protocol. The absorbance was measured at 490 nm. The percentage of LDH release was calculated as follows: (LDH_infected_ − LDH_control_)/ (LDH_total lysis_ − LDH_control_) × 100.

### Cell viability assay

The toxic effects of PG in PMs were examined using a Cell Counting Kit 8 (CCK-8) (Dojindo Laboratories, Kumamoto, Japan), according to the manufacturer’s instructions. PMs were seeded in triplicate in 96-well plates (1 × 10^4^ cells/well), cultured in R-10% medium for 24 h. Then PMs were treated with 15, 30, 60 and 80 μM PG diluted in R-1% medium. Negative control PMs were treated with R-1% medium without the addition of PG. After 24 and 48 h of treatment, 10 μl of CCK-8 reagent was added to each well and incubated for 1.5 h. The absorbance was measured at 450 nm.

### Western blotting

PMs were seeded in 6-well plates at a density of 3 × 10^6^ cells/well, and pre-treated for 1 h with NAC, then infected with *N. caninum* (MOI of 3:1 2:1 or 1:1). In some experiments, PMs were incubated with *N. caninum* for 8 h, then treated with PG at different concentrations. At 24 or 36 h post-infection, the cell lysates and supernatants were collected and assessed by western blotting, as described previously [27]. The following primary antibodies were incubated overnight at 4 °C: anti-mouse IL-1β (p17; 1/1,000; AF-401; R&D, Minneapolis, MN, USA); anti-mouse caspase-1 (p20; 1/1,000; AG-20B-0042; Adipogen, Liestal, Switzerland); anti-NLRP3 (1/1,000; AG-20B-0014, Adipogen); and anti-mouse β-actin (1/2,000; 60008–1; Proteintech, Wuhan, China). Secondary horseradish peroxidase (HRP)-conjugated rabbit anti-goat or anti-mouse IgG (1/5,000; Proteintech) was then added for 1 h at room temperature (RT). Finally, proteins were visualized using an Enhanced chemiluminescence (ECL) Western Blot Detection System (Clinx Science Instruments, Co., Ltd., Shanghai, China).

### Cytokine measurement assay

Active IL-1β in cell supernatants (collected from 3 × 10^6^ PMs) were measured using the mouse IL-1β Ready-Set-Go Kit, and IL-18 in the mice serum were measured using the mouse IL-18 Ready-Set-Go Kit (eBioscience, San Diego, CA, USA), according to the manufacturer’s instructions.

### Real-time quantitative PCR (qPCR)

PMs were seeded in 12-well plates at a density of 1 × 10^6^ cells/well, and pre-treated for 1 h with NAC, then were infected with *N. caninum* (MOI of 1:1). Where required, the PMs were infected *N. caninum* for 4 h, then treated with PG at different concentrations. At 24 h post-infection, the cells were collected. For the *in vivo* assays, mice were intraperitoneally injected with 2.5 × 10^7^ Nc-1 tachyzoites, and peritoneal exudate cells in the peritoneal lavage fluid at the initial infection site, along with heart, liver, spleen, lung, kidney, and brain samples, were collected at day 8 pi All samples were stored at − 20 °C. The parasite replication in cells and tissues were monitored as previously described [35] by real-time qPCR. DNA from 8 × 10^7^ Nc-1 tachyzoites or from infected cells and tissues was extracted using a Genomic DNA Extraction Kit (Tiangen, Beijing, China) according to the manufacturer’s standard protocol. The DNA (200 ng from cells and 500 ng from tissues) samples were subsequently used as the templates in qPCR analyses. A 76-bp fragment of *N. caninum* DNA was amplified using the following primers: forward (5′-ACT GGA GGC ACG CTG AAC AC-3′); reverse (5′-AAC AAT GCT TCG CAA GAG GAA-3′). The number of parasites was determined based on a standard curve obtained using DNA from serial dilutions of *N. caninum* tachyzoites (6.56–6.56 × 10^6^ parasites).

### Immunofluorescence and replication assay

PMs (5 × 10^5^ cells/well) were placed on coverslips in 24-well plates overnight. PMs were challenged with *N. caninum* tachyzoites (MOI of 1:1) for 4 h, then treated with PG. When required, PMs were pre-treated for 1 h with NAC before *N. caninum* infection (MOI of 1:1). At 24 h post-infection, PMs were rinsed 3 times in PBS, fixed with 4% paraformaldehyde for 20 min at RT, washed 3 times with PBS, permeabilized with 0.25% Triton-X-100 in PBS for 10 min, washed in PBS, and blocked with 3% bovine serum albumin (BSA) in PBS for 2 h at RT. The PMs were incubated overnight at 4 °C with primary antibody antiserum against NcSAG1 (1:100; diluted using 1% BSA in PBS; this reacts with *N. caninum*) washed 3 times with PBS, then incubated for 1 h at RT with goat anti-rabbit fluorescein isothiocyanate (FITC)-conjugated secondary antibody (Proteintech) in the dark. The F-actin was labeled with tetramethylrhodamine isothiocyanate (TRITC)-phalloidin (Yeasen, Shanghai, China) for 30 min, and the nuclei were stained with 4′,6-diamidino-2-phenylindole (DAPI; Invitrogen, Carlsbad, CA, USA) for 10 min. Images were captured using an Olympus FV1000 Laser Scanning Confocal Microscope (Japan). The infected cells were observed, the number of parasites in each parasitophorous vacuole was counted and at least 100 parasitophorous vacuoles were counted in the infected group.

### Statistical analysis

Statistical significance was determined by using the Student’s t-test or one-way ANOVA in SPSS 19.0 (SPSS Inc., Chicago, IL, USA), and data are shown as the mean ± standard error of the mean (SE). All graphs were generated using GraphPad Prism 5 (GraphPad Software, Inc., San Diego, CA, USA). All experiments were performed 3 times with 3 technical replicates. *P-*values < 0.05 were considered significant.

## Results

### *Neospora caninum* infection elicited ROS generation in PMs

**Fig. 1:**
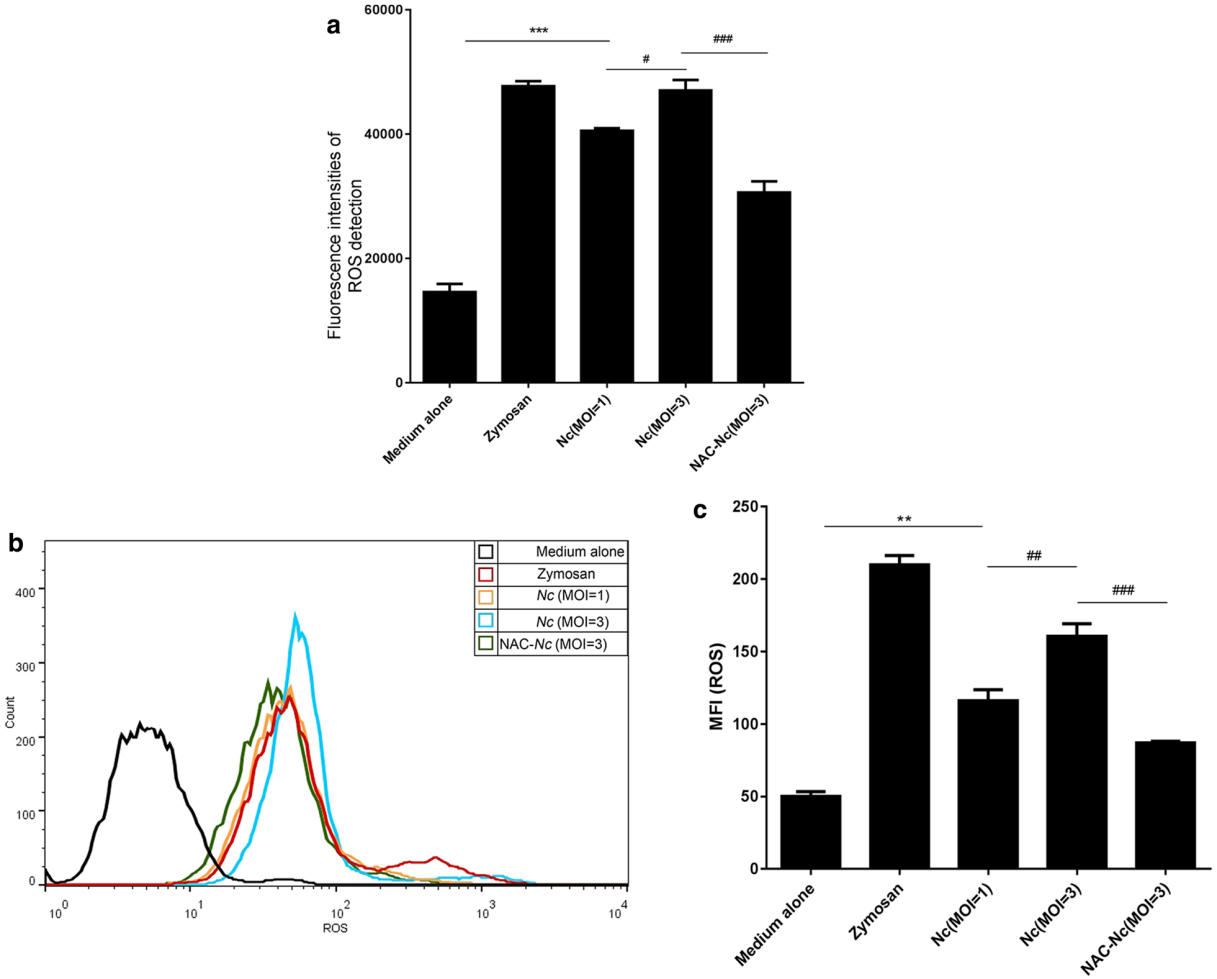
*Neospora caninum* induced ROS generation in PMs. PMs were pre-treated with the ROS inhibitor NAC (5 mM, 1 h), then infected with *N. caninum* at the indicated MOIs for 2 h, and incubated with R-1% medium for 3 h. PMs stimulated with zymosan (1 mg/ml) were used as the positive control. The ROS generation in PMs was determined using the fluorescent probe 2,7 dichlorofluorescein diacetate (DCFH-DA). **a** The ROS generation was assessed by a multifunctional fluorescence microplate reader. **b** The ROS generation was examined by fluorescence activated cell sorter (FACS). **c** The mean fluorescence intensity of ROS was quantified. The data are representative of three independent experiments and shown as the mean ± SE (*n* = 5). *P < 0.05, **P < 0.01, ***P < 0.001 *vs* the negative control; ^#^*P* < 0.05, ^##^*P* < 0.01, ^###^*P* < 0.001 *vs Nc* (MOI = 3)

To investigate whether ROS is generated in *N. caninum*-infected PMs, we detected the level of ROS induced by various MOIs of *N. caninum* tachyzoites in PMs using a DCFH-DA fluorescent probe. Similar with the result of zymosan (positive control)-treated PMs, ROS generation was found in *N. caninum*-infected PMs, but was decreased by the pretreatment of ROS inhibitor NAC (*F*_(4, 10)_ = 366.85, *P* < 0.0001; Fig. 1a). To further confirm our observation, we examined ROS production by measuring the mean fluorescence intensity (MFI) of DCFH-DA. The results further showed that ROS production was significantly triggered by *N. caninum* in a dose-dependent manner. Meanwhile, ROS was significantly inhibited in *N. caninum*-infected PMs with NAC pretreatment (*F*_(4, 10)_ = 125.79, *P* < 0.0001) (Fig. 1b, c). These results suggest that *N. caninum* induces ROS generation in PMs.

### ROS inhibitor NAC attenuated *N. caninum-*induced NLRP3 inflammasome activation and increased *N. caninum* proliferation in PMs

**Fig. 2:**
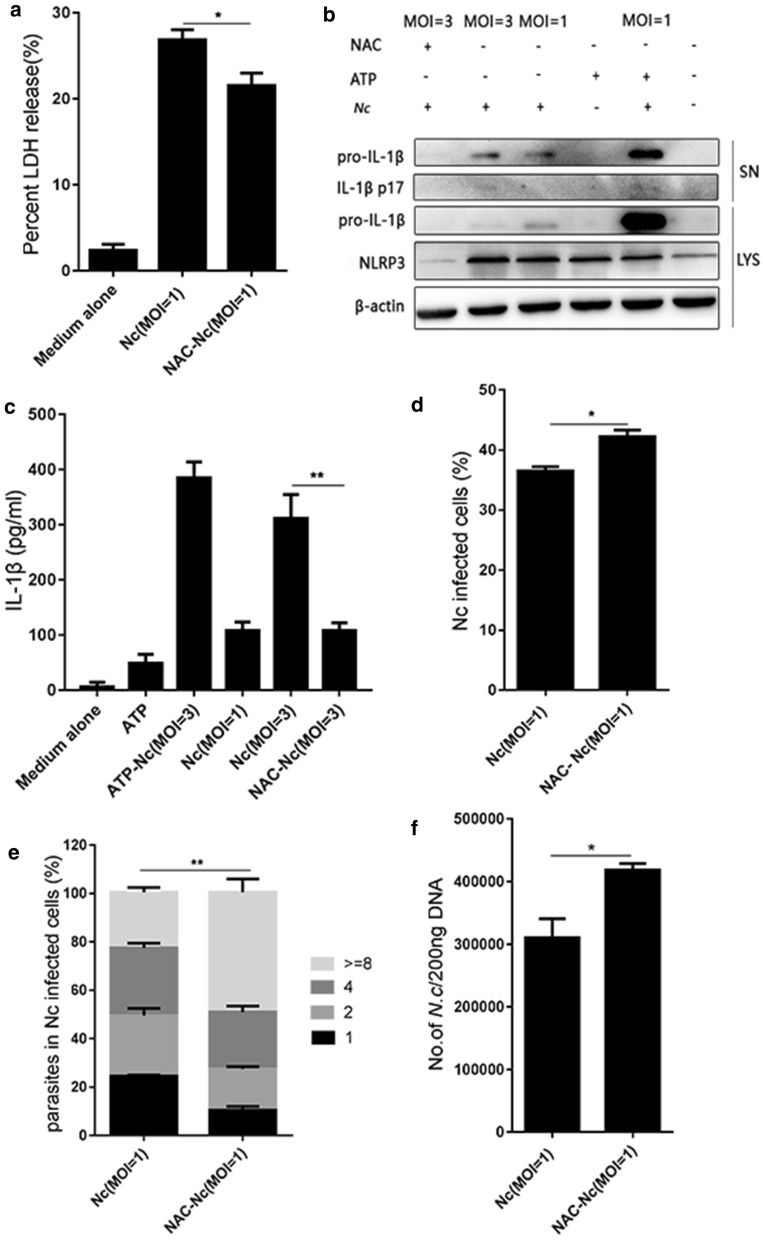
Roles of the inhibitor NAC in regulating NLRP3 inflammasome and parasite proliferation during *N. caninum* infection. PMs were pre-treated with or without NAC (5 mM; 1 h) and infected with *N. caninum* (MOI of 3:1 or 1:1, parasite: cell; 8 h). ATP (5 mM; 30 min) added to the *N. caninum*-infected cells was used as a positive control. At 24 h post-infection, the cell lysates and supernatants were collected. **a** Cell death was monitored by measuring LDH release in supernatants. **b** NLRP3 expression and active IL-1β (p17) was assessed by western blotting. **c** IL-1β production in supernatants was measured by ELISA. PMs were pre-treated with or without NAC (5 mM; 1 h) and then infected with *N. caninum* at a MOI of 1 for 4 h. At 24 h post-infection, the cells were collected. **d** Percentage of *N. caninum* infected cells was counted by fluorescence confocal microscopy. **e** Quantification of parasites in parasitophorous vacuoles were monitored by fluorescence confocal microscopy. **f** Number of *N. caninum* in infected PMs was assessed by quantitative PCR. The data are shown as the mean ± SE from three independent experiments. *P < 0.05, **P < 0.01, and ***P < 0.001 *vs* the NAC group

To elucidate whether the ROS participates in NLRP3 inflammasome activation and the control of the parasite burden in *N. caninum*-infected PMs, PMs were pre-treated with the ROS inhibitor NAC, then the secretion of active IL-1β and the expression of NLRP3 were assessed by western blotting, inflammasome-dependent cell death was detected using an LDH assay. In addition, the parasite burden was monitored by qPCR using total DNA (200 ng) from *N. caninum*-infected PMs, and quantification of parasites in parasitophorous vacuoles of *N. caninum*-infected cells was carried out using confocal microscopy. Results showed that *N. caninum* induced inflammasome-dependent LDH release in PMs, while NAC significantly decreased LDH release (*F*_(2, 6)_ = 125.7, *P* < 0.0001; Fig. 2a). Additionally, NAC abrogated *N. caninum*-induced NLRP3 expression in cells and active IL-1β secretion in the supernatants (active IL-1β: *F*_(5, 12)_ = 141.92, *P* < 0.0001) (Fig. 2b, c). Moreover, the percentage of *N. caninum*-infected PMs (assessed by confocal microscopy) was significantly increased in the NAC-pre-treated group (*t*_(4)_ = 3.49, *P* = 0.0251; Fig. 2d), and the parasite burden (assessed by confocal microscopy and qPCR) was also higher in the NAC-pre-treated group than that in the *N. caninum* infection-only group (Fig. 2e: *t*_(4)_ = 8.078, *P* = 0.0013; Fig. 2f: *t*_(4)_ = 3.384, *P* = 0.0277). The results showed that NLRP3 inflammasome activation was inhibited and the parasite burden was increased by NAC pretreatment in *N. caninum*-infected PMs. These data suggest that ROS plays a very important role in regulating NLRP3/ IL-1β and the control of intracellular parasite replication in *N. caninum*-infected PMs.

### ROS inducer PG inhibited *N. caninum* replication by promoting NLRP3 inflammasome activation *in vitro*

**Fig. 3:**
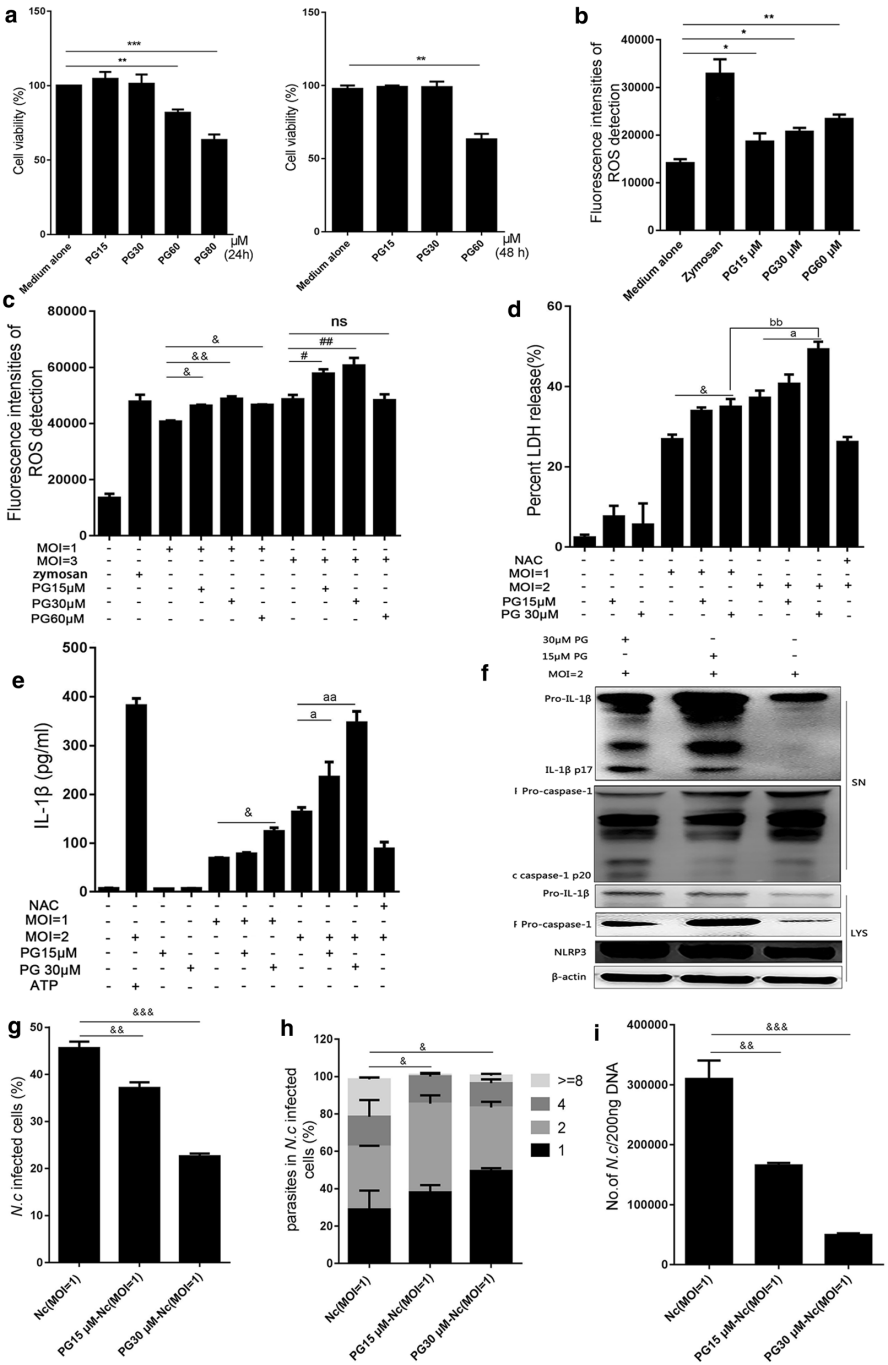
PG inhibited *N. caninum* replication by increasing NLRP3 inflammasome activation *in vitro*. PMs were treated with PG at various concentrations (15, 30, 60 and 80 μM) for 24 or 48 h. **a** The cell viability of PMs was determined by CCK-8 assays. **b** PMs were treated with PG (15 or 30 or 60 μM) for 3 h, PMs stimulated with zymosan (1 mg/ml; 1 h) was used as positive controls, ROS production in PMs was assessed using a multifunctional fluorometric reader. PMs were infected with *N. caninum* at the indicated MOIs for 2 h, and incubated with or without PG for 3 h. **c** ROS production was detected by a multifunctional fluorometric reader. PMs were infected with *N. caninum* at the indicated MOIs for 8 h, then incubated with or without PG. At 36 h post-infection, **d** cell death was monitored by measuring LDH activity in the supernatants, **e** IL-1β in supernatants was measured using ELISA, **f** NLRP3, IL-1β (p17) and caspase-1 (p20) were monitored by western blotting. PMs were infected with *N. caninum* at the indicated MOIs for 4 h, then incubated with or without PG. At 24 h post-infection, percentage of *N. caninum* infected cells (**g**) and quantification of parasites (**h**) in parasitophorous vacuoles were monitored by fluorescence microscopy. **i** Number of *N. caninum* in infected PMs was determined by qPCR. The data are shown as the mean ± SE from three independent experiments. *P < 0.05, **P < 0.01, ***P < 0.001 *vs* the cell; ^&^*P* < 0.05, ^&&^*P* < 0.01, ^&&&^*P* < 0.001 *vs* Nc (MOI = 1); ^#^*P* < 0.05, ^##^*P* < 0.01 *vs* Nc (MOI = 3); ^a^*P* < 0.05, ^aa^*P* < 0.01 *vs* Nc (MOI = 2); ^bb^*P* < 0.01 *vs* PG30 μM-Nc (MOI = 1)

To further explore the relationship between ROS and the NLRP3 inflammasome response to *N. caninum* infection in PMs, *N. caninum*-infected PMs were treated with the ROS inducer PG. We first analyzed the cytotoxicity of PG by treating PMs with PG at various concentrations (15, 30, 60 and 80 μM) for 24 or 48 h and then performing the CCK-8 assay. The results showed cell viability was not inhibited by PG treatment at concentrations of 15 or 30 μM, while the opposite effect was observed at concentrations of 60 or 80 μM (24 h: *F*_(4, 10)_ = 57.69, *P* < 0.0001; 48 h: *F*_(3, 8)_ = 187.38, *P* < 0.0001; Fig. 3a), but ROS generation in PMs was significantly increased in a dose-dependent manner (*F*_(4, 10)_ = 93.05, *P* < 0.0001; Fig. 3b). The PG treatment can greatly increase *N. caninum*-triggered intracellular ROS production compared with the infection-only group by the multifunctional fluorescence microplate reader (*F*_(9, 20)_ = 363.87, *P* < 0.0001; Fig. 3c). LDH release can be induced by *N. caninum* and indicate inflammasome-dependent cell death, and *N. caninum* induced LDH releases were significantly increased after PG treatment in a dose-dependent manner (*F*_(9, 20)_ = 143.81, *P* < 0.0001; Fig. 3d). In addition, in accord with the positive control (ATP) group, *N. caninum* induced active IL-1β secretions were also greatly increased by PG treatments (*F*_(10, 22)_ = 555.95, *P* < 0.0001; Fig. 3e). The western-blot results show that NLRP3, cleavage of caspase-1 and active IL-1β were significantly increased after PG treatment of *N. caninum*-infected PMs for 36 h; the effects of PG were most notable at 30 μM (Fig 3f). These results suggest that *N. caninum*-induced NLRP3 inflammasome activation was upregulated by the ROS inducer PG.

To investigate whether PG-regulated NLRP3 inflammasome activation also plays a role in eliminating *N. caninum* in PMs, *N. caninum*-infected PMs were treated with or without PG, parasites in parasitophorous vacuoles of *N. caninum*-infected PMs were quantified by confocal microscopy. The results showed that the percentage of *N. caninum* infected cells was decreased in the PG-treated group in a dose-dependent manner (*F*_(2, 6)_ = 226.99, *P* < 0.0001; Fig. 3g), and the parasite number in parasitophorous vacuoles of PMs was also decreased in the PG-treated group compared with the infection-only group (*F*_(2, 6)_ = 230.48, *P* < 0.0001; Fig. 3h). Meanwhile, the number of *N. caninum* in PMs was further assessed by qPCR, and the parasite burden in PMs was indeed significantly decreased in the PG-treated group in a dose-dependent manner (*F*_(2, 6)_ = 55.721, *P* < 0.0001; Fig. 3i). These results indicate that PG can inhibit the replication of *N. caninum* in PMs.

### NLRP3 deletion blocked ROS-mediated inflammasome activation in *N. caninum*-infected PMs

**Fig. 4:**
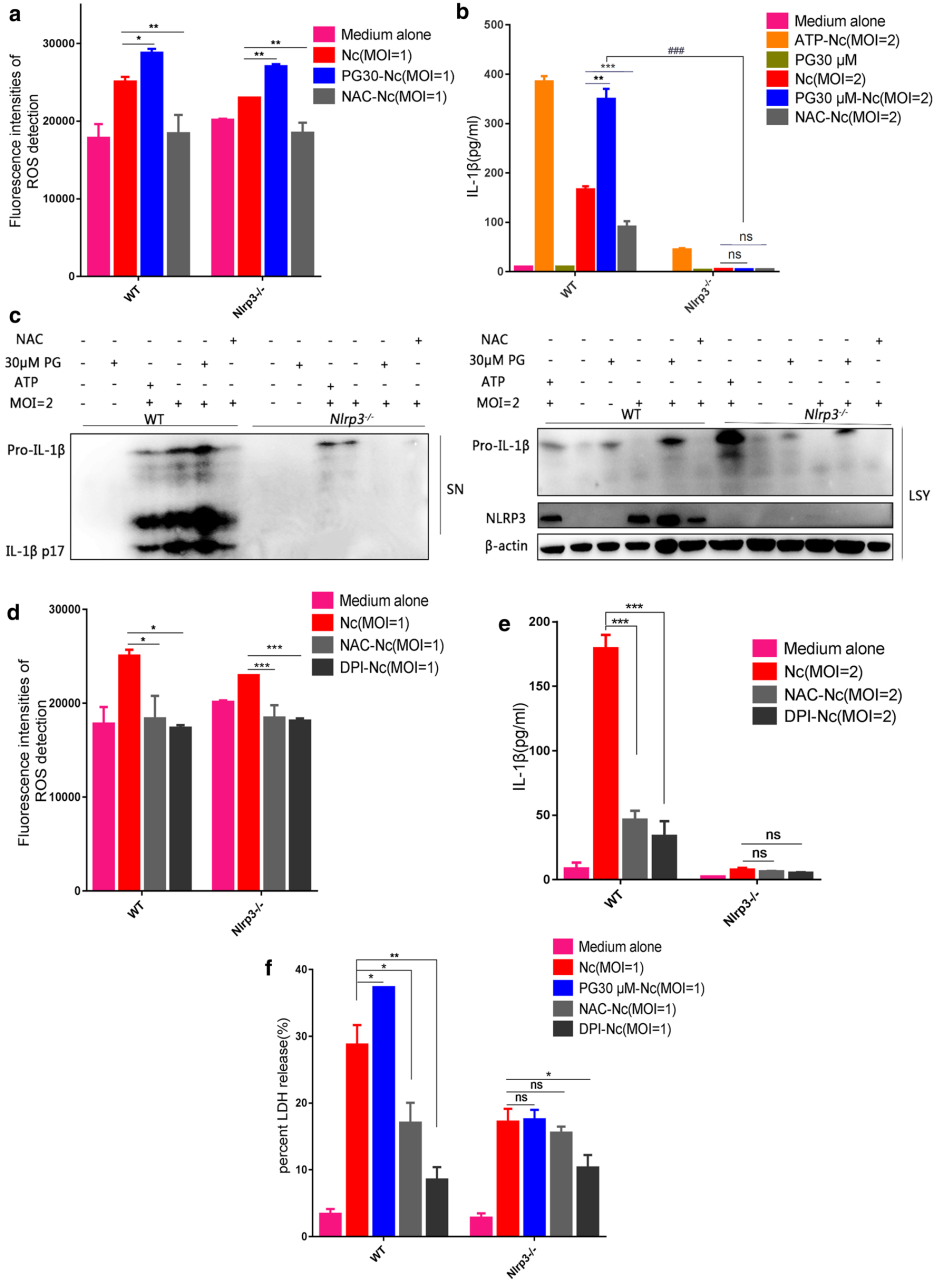
NLRP3 deletion blocked ROS-induced inflammasome activation during *N. caninum* infection *in vitro*. WT and *Nlrp3*^*−/−*^ PMs were pre-treated with or without the ROS inhibitor NAC (5 mM, 1 h), then infected with *N. caninum* at an MOI of 1 for 2 h, infected PMs were incubated with or without PG at post infection for 3 h. **a** ROS production was assessed using a multifunctional fluorometric reader. WT and *Nlrp3*^*−/−*^ PMs were pre-treated with or without the ROS inhibitor NAC (5 mM, 1 h), then infected with *N. caninum* at an MOI of 2 for 8 h, and incubated with or without PG. ATP (5 mM; 30 min) added to the *N. caninum*-infected cells was used as a positive control. At 36 h post-infection, IL-1β in supernatants was detected by ELISA (**b**), IL-1β (p17) and NLRP3 expression were detected by western blotting (**c**). WT and *Nlrp3*^*−/−*^ PMs were pre-treated with or without the ROS inhibitor NAC (5 mM, 1 h) or DPI (10 μM, 2 h), and infected with *N. caninum* at an MOI of 1 for 2 h, then cultured in fresh R-1% medium for 3 h, ROS production was assessed using a multifunctional fluorometric reader (**d**); or infected with *N. caninum* at the indicated MOIs for 8 h, and incubated with or without PG, at 36 h post-infection, IL-1β in supernatants was detected by ELISA (**e**). **f** Percent LDH release is measured by the LDH assay kit. The data are shown as the mean ± SE from three independent experiments. *P < 0.05, **P < 0.01, ***P < 0.001 *vs* the Nc group in WT PMs or *Nlrp3*^*−/−*^ PMs; ^###^*P* < 0.001 *vs* PG30-Nc (MOI = 2) in *Nlrp3*^*−/−*^ PMs

To explore whether NLRP3 is one of the primary factors underlying ROS-mediated inflammasome activation, we studied the effects of the ROS inhibitor NAC and the ROS inducer PG on inflammasome activation in *Nlrp3*^*−/−*^ PMs. We found that the intracellular ROS was increased by PG or attenuated by NAC in *N. caninum*-infected WT and *Nlrp3*^*−/−*^ PMs (WT: *F*_(3, 8)_ = 29.07, *P* = 0.0001; *Nlrp3*^*−/−*^: *F*_(3, 8)_ = 114.732, *P* < 0.0001; Fig. 4a). NLRP3 was essential for *N. caninum*-induced IL-1β secretion [36]. We found that NLRP3 expression in cell lysates and active IL-1β secretion in supernatants of *N. caninum*-infected WT PMs were greatly increased by PG treatment. In contrast, NAC significantly decreased NLRP3 expression and active IL-1β production in WT PMs (active IL-1β: WT: *F*_(5, 12)_ = 929.66, *P* < 0.0001; *Nlrp3*^*−/−*^: *F*_(5, 12)_ = 263.71, *P* < 0.0001) (Fig. 4b, c). However, compared with the WT group, active IL-1β production was inhibited in *Nlrp3*^*−/−*^ *N. caninum*-infected PMs, and NAC or PG treatment also failed to alter active IL-1β secretion in *Nlrp3*^*−/−*^ PMs (Fig. 4b, c). These results indicate that ROS depends on NLRP3 to activate the inflammasome in *N. caninum*-infected PMs.

To further explore whether *N. caninum* induced NADPH-dependent ROS production to mediate NLRP3 inflammasome activation, PMs were pre-treated with the NADPH oxidase inhibitor DPI. Similar with NAC, the intracellular ROS was markedly attenuated by DPI in *N. caninum*-infected WT and *Nlrp3*^*−/−*^ PMs (WT: *F*_(3, 8)_ = 11.92, *P* = 0.0030; *Nlrp3*^*−/−*^: *F*_(3, 8)_ = 42.45, *P* < 0.0001; Fig. 4d). Expectedly, active IL-1β production was also significantly decreased by DPI in *N. caninum*-infected WT PMs. Upon knockout of NLRP3, DPI did not alter the IL-1β secretion of *N. caninum* infected PMs compare to the infection-only PMs (WT: *F*_(3, 8)_ = 82.94, *P* < 0.0001; *Nlrp3*^*−/−*^: *F*_(3, 8)_ = 1.265, *P* = 0.3500; Fig. 4e). In addition, we detected the influences of NAC, DPI and PG on the LDH release in *N. caninum* infected PMs. The results found that inducer PG significantly increased the LDH release in *N. caninum* infected WT PMs, but LDH release was greatly reduced by inhibitors NAC and DPI. As expected, NAC and PG did not influence the LDH release in the *Nlrp3*^*−/−*^ *N. caninum* infected PMs (WT: *F*_(4, 10)_ = 39.82, *P* < 0.0001; *Nlrp3*^*−/−*^: *F*_(4, 10)_ = 22.53, *P* < 0.0001; Fig. 4f). The release of ROS, IL-1β, and LDH in DPI-pre-treated WT PMs were lower than that in NAC pre-treatment. These phenomena further suggest that NADPH-dependent ROS is an upstream signal that activates NLRP3 inflammasome during *N. caninum* infection.

### PG resisted *N. caninum* infection by regulating NLRP3 inflammasome pathway

**Fig. 5:**
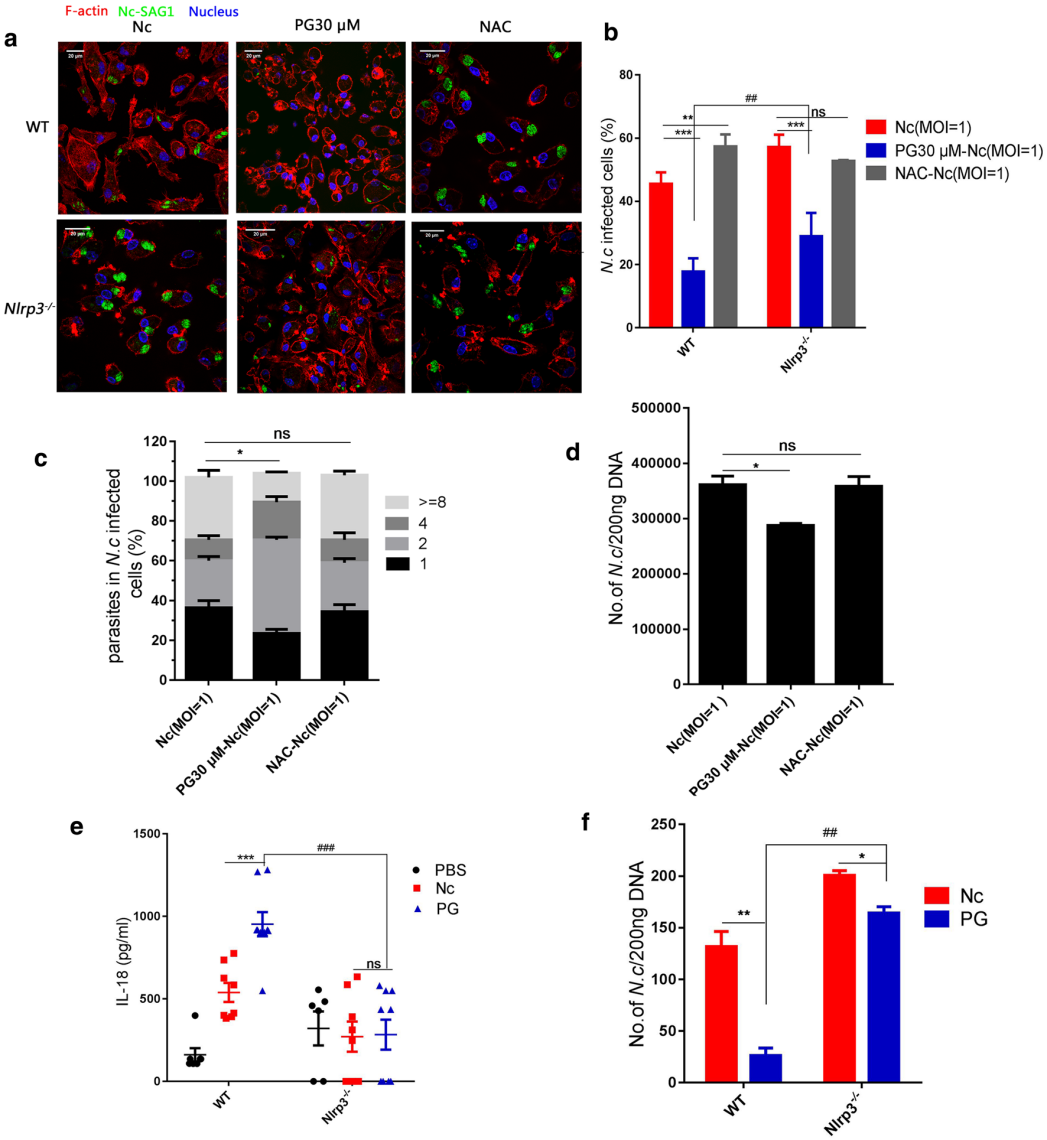
PG regulated ROS-NLRP3 pathway in resisting *N. caninum* infection. WT and *Nlrp3*^*−/−*^ PMs were pre-treated with or without NAC (5 mM, 1 h), then infected with *N. caninum* an MOI of 1 for 4 h, and *N. caninum*-infected PMs were incubated with or without PG (30 μM). The PMs were collected at 24 h post-infection. **a** PMs were stained with polyclonal antiserum against NcSAG1, which reacts with *N. caninum* tachyzoites. The F-actin was labeled with TRITC-phalloidin and the nuclei was stained with DAPI for confocal microscopy observation. **b** Percentage of *N. caninum* infected cells was monitored by fluorescence microscopy. **c** Quantification of parasites in vacuoles in *Nlrp3*^*−/−*^ PMs were monitored by fluorescence microscopy. **d** Number of parasites in *Nlrp3*^*−/−*^ PMs were detected by quantitative PCR. WT and *Nlrp3*^*−/−*^ mice were intraperitoneally infected with 2.5 × 10^7^ *N. caninum* tachyzoites or PBS (0.5 ml). After 2 days, the infected groups were injected with PG (75 mg/kg, in 0.5 ml PBS) or PBS (0.5 ml). Serum and peritoneal exudate cells were collected at day 8. **e** IL-18 in serum was measured by ELISA. **f** Parasite burdens in peritoneal exudate cells was detected by quantitative PCR. The data are shown as the mean ± SE from three independent experiments. *P < 0.05, **P < 0.01, ***P < 0.001 *vs* the Nc group in WT PMs or *Nlrp3*^*−/−*^ PMs; ^##^*P* < 0.01, ^###^*P* < 0.001 *vs* PG30-Nc (MOI = 2) in *Nlrp3*^*−/−*^ PMs

To evaluate the influence of ROS-mediated NLRP3-dependent inflammasome on the parasite burden, *N. caninum*-infected WT PMs or *Nlrp3*^*−/−*^ PMs were pre-treated with the ROS inhibitor NAC or treated with the ROS inducer PG. The results showed that the more *N. caninum* infected cells and parasite burdens in *Nlrp3*^*−/−*^ PMs were observed than in WT PMs (Fig. 5a). The percentage of *N. caninum* infected WT PMs (based on confocal microscopy) was significantly decreased by PG treatment, but greatly increased by NAC pre-treatment compared with the infection-only WT PMs. In contrast, no significant difference in the percentage of *N. caninum* infected cells was found between NAC pre-treated *Nlrp3*^*−/−*^ PMs and the infection-only *Nlrp3*^*−/−*^ PMs, though PG did cause a slight decrease in *Nlrp3*^*−/−*^ PMs (WT: *F*_(2, 6)_ = 94.42, *P* < 0.0001; *Nlrp3*^*−/−*^: *F*_(2, 6)_ = 48.37, *P* = 0.0002; Fig. 5b). To further determine the effects of the NAC and the PG on the parasite burden of *Nlrp3*^*−/−*^ PMs, we counted the parasite number in parasitophorous vacuoles of *Nlrp3*^*−/−*^ PMs using confocal microscopy and analyzed the number of *N. caninum* in *Nlrp3*^*−/−*^ PMs by qPCR. The results showed that there was no significant difference in parasite burdens between the NAC-pre-treated and infection-only groups, but the PG-treated group exhibited a slight decrease in parasite burden (Fig. 5c: *F*_(2, 6)_ = 58.83, *P* = 0.0001; Fig. 5d: *F*_(2, 6)_ = 9.765, *P* = 0.0130). These results show that ROS-mediated NLRP3-dependent inflammasome activation plays a key role in controlling *N. caninum* infection, and the PG can inhibit parasite replication in *N. caninum*-infected PMs mainly by promoting NLRP3 inflammasome activation and other mechanism.

To further explore the role of PG-induced NLRP3 inflammasome activation in inhibiting *N. caninum* infection *in vivo*, WT and *Nlrp3*^*−/−*^ mice were infected with *N. caninum* and then treated with PG. Inflammasome-mediated IL-18 secretion in serum and parasite burdens in peritoneal exudate cells were measured. The results showed that serum IL-18 in PG-treated WT mice was obviously higher than in infection-only WT mice, but there was no significant difference in IL-18 production between *N. caninum-*infected *Nlrp3*^*−/−*^ mice with or without PG treatment, and IL-18 production in *Nlrp3*^*−/−*^ mice induced by PG treatment was lower than the production in the corresponding WT mice (WT: *F*_(2, 12)_ = 76.96, *P* < 0.0001; *Nlrp3*^*−/−*^: *F*_(2, 12)_ = 0.615, *P* = 0.5566; Fig. 5e). After PG treatment, parasite burdens in peritoneal exudate cells in WT mice were significantly decreased. In addition, parasite burdens in peritoneal exudate cells of *Nlrp3*^*−/−*^ mice was significantly increased than in WT mice. With PG treatment the parasite burden was greatly decreased in both WT and *Nlrp3*^*−/−*^ mice, and PG treatment had a more significant inhibiting effect on WT mice than in *Nlrp3*^*−/−*^ mice (WT: *t*_(4)_ = 11.26, *P* = 0.0004; *Nlrp3*^*−/−*^: *t*_(4)_ = 8.416, *P* = 0.0011; Fig. 5f). These results further confirm the findings of the *in vitro* experiments that the effects of PG on regulating NLRP3 inflammasome activation and indicate that PG plays a vital role in controlling *N. caninum* infection in both NLRP3 dependent manner and other mechanism *in vivo*.

### Roles of the PG in controlling *N. caninum* proliferation and protecting host

**Fig. 6:**
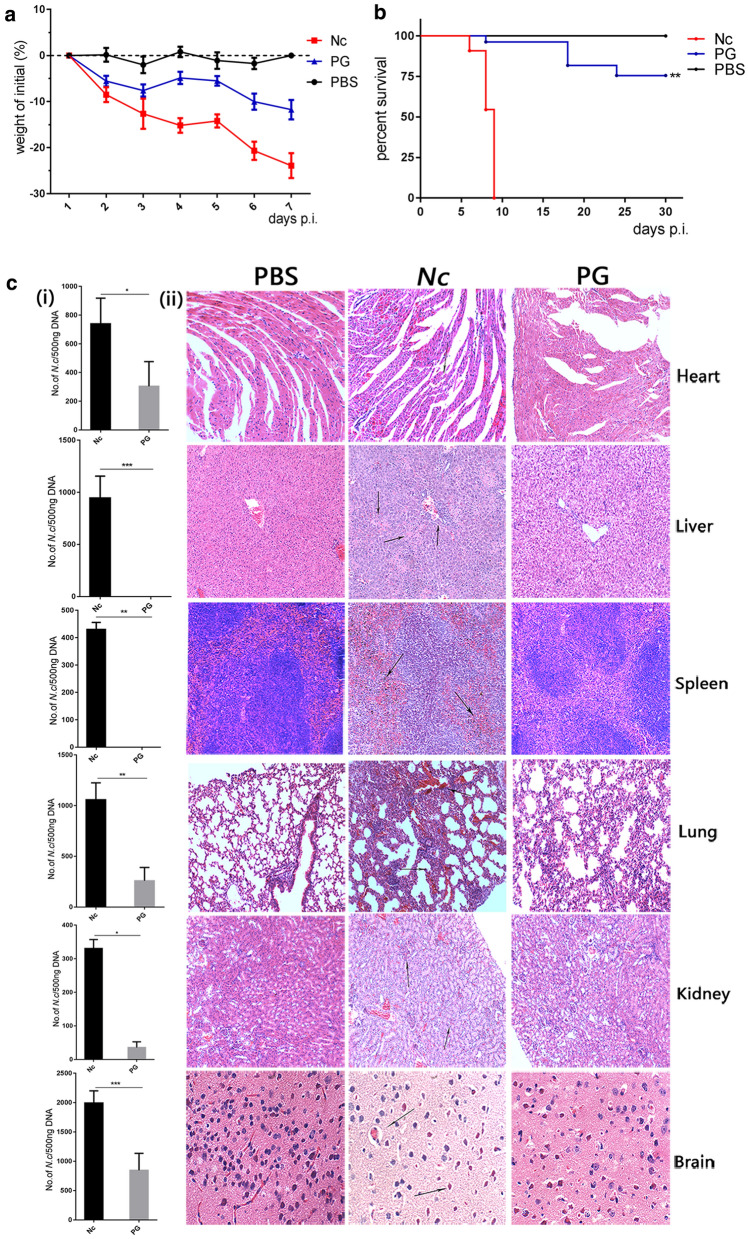
Roles of the PG in controlling *N. caninum* proliferation and protecting host. WT mice were intraperitoneally infected with 2.5 × 10^7^ *N. caninum* tachyzoites or PBS (0.5 ml) for 2 days and then the infected groups were injected with PG (75 mg/kg, in 0.5 ml PBS) or PBS (0.5 ml). Each group of mice were monitored daily. **a** Weight change in mice was recorded for 7 days. **b** Survival of mice was monitored for 30 days. **c**(i) Parasite burdens were measured by quantitative PCR of total DNA (500 ng). **c**(ii) Histopathological analysis of heart, liver, spleen, lung, kidney and brain tissues of WT mice were conducted with H&E staining at day 8 post-infection. Heart, liver, spleen, lung, and kidney tissues were examined at a magnification of 100×, and the brain tissue was examined at a magnification of 400×. The data are shown as the mean ± SE from three independent experiments. *P < 0.05, **P < 0.01, ***P < 0.001 *vs* the Nc group

To further investigate whether the ROS-NLRP3 axis could be a candidate pathway to identify drugs for the treatment of neosporosis and explore the effects of PG on *N. caninum*-infected mice. The WT mice were intraperitoneally injected with or without 2.5 × 10^7^ *N. caninum* tachyzoites, then *N. caninum-*infected mice were intraperitoneally injected with or without PG. Weight, survival time, parasite burdens and pathological changes in tissues were monitored. The results showed that the weight loss caused by *N. caninum* infection was decreased in PG group compared with the infection-only group (Fig. 6a). In addition, the infection-only group had a lower survival rate, and none of the mice survived after the ninth day post-infection. However, PG treatment can greatly increase the survival rate of infected mice when compare with the infection-only group (*t*_(4)_ = 6.047, *P* = 0.0038; Fig. 6b).

To investigate the roles of PG in controlling parasite replication and dissemination in tissues, samples from the heart, the liver, the spleen, the lung, the kidney and the brain of mice were collected to assess parasite burdens and histopathological changes. Parasite burdens in these tissues were significantly decreased in the PG-treated group compared with the infection-only group on day 8 pi (heart: *t*_(4)_ = 3.031, *P* = 0.0387; liver: *t*_(4)_ = 7.801, *P* = 0.0015; spleen: *t*_(4)_ = 28.65, *P* < 0.0001; lung: *t*_(4)_ = 6.502, *P* = 0.0029; kidney: *t*_(4)_ = 16.22, *P* < 0.0001; and brain: *t*_(4)_ = 5.589, *P* = 0.0050; Fig. 6c-(i)). Histopathological examination showed that heart, liver, spleen, lung, kidney, and brain tissues from the infection-only group had severe inflammatory responses with hemorrhage, vascular phenomena, leukocyte margination, interstitial thickening, and necrotic foci when compared to that in PBS group. These findings included hemorrhagic symptoms in the heart tissues, degenerative changes, and necrosis in liver tissues. Hemorrhage and erythrocytes in the spleen white pulp were increased, while the damage to the heart, liver and spleen were considerably mitigated in the PG treatment group, and only a small amount of damage was observed in the liver and spleen. Alveolar wall thickening, neutrophil infiltration, and alveolar hemorrhage were exhibited in the lungs in the infection-only group, whereas only slight pathological symptoms were observed in the PG-treated group. The brain in infected mice showed degeneration and necrosis of neurons, severe meningitis, and severe perivascular inflammation, whereas milder histopathological changes were observed in brains of the PG-treated mice (Fig. 6c-(ii)). Taken together, these results indicate that PG has significant protecting effects on the host by decreasing the death rate, weight loss, parasite burdens in tissues and the tissue lesions. These indicate that ROS inducer PG could be a potential drug for treating neosporosis and the ROS-mediated NLRP3 pathway could be used to identify further candidate drugs for the treatment of neosporosis.

## Discussion

*Neospora caninum* can invade and replicate in PMs and induce immune responses [8]. The innate immune response plays a key role in controlling pathogen infection, and mediates an appropriate adaptive immune response to promote and strengthen the elimination of invasive parasites, as well as build immunological memory to protect against reinfection [37]. IL-1β is a marker of NLRP3 inflammasome activation and essential for cell defense in response to parasite infection. Previous studies have reported that *N. caninum* infection can induce cleavage of caspase-1 and IL-1β secretion in PMs [8] and bone marrow-derived macrophages [36] and bovine macrophages [38], However, the molecular mechanism of *N. caninum*-induced NLRP3 inflammasome activation was still unclear. In this study, we demonstrate that NLRP3 inflammasome activation is triggered by *N. caninum*-induced ROS production, and the ROS inducer PG controls *N. caninum* infection mainly by regulating NLRP3-dependent inflammasome activation *in vitro* and *in vivo*.

NLRP3 inflammasome activation requires two signals. The first signal is provided by the activation of nuclear factor NF-κB, which upregulates NLRP3, pro-IL-1β and pro-IL-18. The second signal is provided by various DAMPs or PAMPs, and leads to the formation of inflammasome complex [39, 40]. The second signal stimuli include numerous pathogens such as bacteria, viruses, fungi, and their components, ATP, pore-forming toxins, and particulate crystals and aggregates [41–43]. ROS generation is regarded as one of the molecular mechanisms underlying NLRP3 inflammasome activation [19]. ROS production is one of the most important factors for various biological functions, including proliferation, differentiation, immune response and cell survival [44]. Currently, ROS-induced NLRP3 inflammasome activation remains controversial [45]. Many studies have reported that the NLRP3 inflammasome in *Trypanosoma cruzi*-infected macrophages is inhibited by the inhibition of ROS [22], but Bortolucci’s group demonstrated that *Trypanosoma cruzi*-induced NLRP3 activation is independent on ROS generation [13]. *Leishmania donovani*-mediated ROS generation inhibits NLRP3 inflammasome activation in RAW 264.7 cells [46]. These differences in ROS-mediated NLRP3 inflammasome activation differ among the protozoan parasites or cell types. Therefore, it is necessary to explore ROS-mediated NLRP3 inflammasome activation in *N. caninum*-infected PMs. In the present study, we found that ROS generation and NLRP3 inflammasome activation were induced in dose-dependent manners by *N. caninum* infection in PMs.

ROS production is related to the occurrence of respiratory burst in macrophages, it is a process that depends on NADPH oxidase activation to promote the rapid increase of superoxide free radicals and H_2_O_2_ in early recognition of pathogens [47]. Cell death and expressions of NLRP3, caspase-1 and IL-1β induced by nicotine in endothelial cells were decreased by NAC pretreatment [48]. IL-1β secretion in THP1 cells was diminished by the NADPH oxidase inhibitor DPI and the ROS inhibitor NAC [27]. In our study, similar results were observed in NAC or DPI pre-treated WT PMs, *N. caninum*-induced releases of ROS, IL-1β and LDH were significantly decreased in both DPI group and NAC group. These results indicate that *N. caninum*-induced NADPH oxidase-dependent ROS generation triggers NLRP3 inflammasome activation in PMs. The ROS inhibitors also have various side effects, but they are still effective in demonstrating the effects of ROS on NLRP3 inflammasome [20]. And whether mitochondrial ROS is responsible for NLRP3 inflammasome activation during *N. caninum* infection needs further study. IL-1β secretion was induced in *N. caninum*-infected PMs, at the MOI of 3 for 24 h, and *N. caninum* infection of PMs induced significant IL-1β secretion in time- and dose-dependent manners [8], IL-1β secretion of PMs infected with *N. caninum* at an MOI of 3 for 24 h was lower. But the cell death of PMs infected with *N. caninum* at an MOI of 3 for 36 h was greatly higher (data not shown), so we chose an MOI of 2 for 36 h to establish the *N. caninum* infection model of PMs in activating NLRP3 inflammasome, and an MOI of 1 for 24 h to establish the *N. caninum* infection model of PMs in monitoring parasite burdens. A previous study has found that *N. caninum*-induced inflammasome activation is mainly dependent on NLRP3 [36]. In this study, ROS generations induced by PG or attenuated by NAC or DPI, which did not completely alter the inflammasome activities in *N. caninum*-infected *Nlrp3*^*−/−*^ PMs. This indicates that ROS-mediated inflammasome activation mainly depends on NLRP3 during *N. caninum* infection. Interestingly, DPI still inhibited LDH release in *Nlrp3*^*−/−*^ cells, probably by other mechanisms [30]. Additionally, ROS-NLRP3 inflammasome activity was observed in *N. caninum*-infected mice. These results suggest that ROS depends on NLRP3 to activate the inflammasome during *N. caninum* infection *in vitro* and *in vivo*, and ROS is an activator of *N. caninum*-induced NLRP3 inflammasome.

The NLRP3 inflammasome plays an essential role in controlling pathogenic microbial infections [8, 49]. *Toxoplasma gondii*-induced NLRP3 inflammasome activation inhibits parasite proliferation in macrophages and human small intestinal epithelial cells [50, 51]. *Neospora caninum-*induced NLRP3 inflammasome activation is beneficial for parasites elimination in PMs and mice [8, 36]. *Neospora caninum* can induce NF-κB activation [11], this signal was used as a first signal, and ROS inducer PG was applied as a second signal to enhance NLRP3 inflammasome activation in this study. Weight loss, mortality and histopathological lesions are increased in *N. caninum-*infected WT mice [36, 52], these are important data to assess *N. caninum* infection in mice. We chose 75 mg/kg PG for the *in vivo* study, because this dose is relatively safe for mice [32, 33]. Here, we found that PG had a good effect on decreasing mortality and weight loss, and the parasite burden in tissues was also significantly decreased after PG treatment when compared with the infection-only WT mice. In addition, histopathological lesions were also alleviated in the PG-treated group, similar to the effect of licochalcone A that induces ROS generation to combat *T. gondii in vivo* [53], and kojic acid promotes superoxide anion (O_2_^•^) generation and exhibits significant direct and indirect anti-*Toxoplasma* activity [54]. Previous studies only reported that PG inhibits cancer cell growth [55–57] and enhances cell death by increasing O_2_^·‑^ levels [58, 59]. To the best of our knowledge, our study is the first to indicate that ROS inducer PG promotes NLRP3-dependent inflammasome activation to inhibit parasite proliferation *in vivo* and *in vitro*. In addition, PG also has a NLRP3-independent manner in controlling *N. caninum* infection, but the underlying mechanisms require further evidence [22, 60]. Taken together, ROS-mediated NLRP3 inflammasome activation could be a candidate pathway for identifying new drugs for the treatment of neosporosis, and PG may be a new drug for the treatment of neosporosis, but further research on PG in bovine species is still needed.

## Conclusions

We demonstrate the relationships between ROS, the NLRP3 inflammasome, and *N. caninum* infection. *Neospora caninum* can induce NADPH-dependent ROS production in PMs, and ROS-mediated NLRP3 inflammasome activation participated in host response against *N. caninum* infection. Furthermore, we found that ROS inducer PG promoted NLRP3 inflammasome activation to increase host resistance during *N. caninum* infection. This study indicates that ROS-mediated NLRP3 inflammasome has implications for providing a new solution for the treatment of neosporosis in the future, and enriches our knowledge of the molecular mechanism for host innate immune response and host defense during *N. caninum* infection.

## Data Availability

All data generated or analyzed during the present study are included in this published article.

## Abbreviations

BSA: bovine serum albumin
qPCR: quantitative polymerase chain reaction
ELISA: enzyme-linked immunosorbent assay
FBS: fetal bovine serum
CCK-8: cell counting kit-8
IL-1β: interleukin-1β
DAPI: 4′,6-diamidino-2-phenylindole
TRITC: tetramethylrhodamine isothiocyanate
SN: supernatants
LYS: cell lysates
LDH: lactate dehydrogenase
MOI: multiplicities of infection
PBS: phosphate buffer saline
PBST: phosphate-buffered saline with tween-20
PCR: polymerase chain reaction
SE: standard error of the mean
